# Roles and Mechanisms of Herbal Medicine for Diabetic Cardiomyopathy: Current Status and Perspective

**DOI:** 10.1155/2017/8214541

**Published:** 2017-10-24

**Authors:** Jinfan Tian, Yingke Zhao, Yanfei Liu, Yue Liu, Keji Chen, Shuzheng Lyu

**Affiliations:** ^1^Department of Cardiology, Beijing Anzhen Hospital, Capital Medical University, Beijing 100029, China; ^2^Cardiovascular Disease Centre, Xiyuan Hospital, China Academy of Chinese Medical Sciences, Beijing 100091, China; ^3^School of Chinese Medicine, Li Ka Shing Faculty of Medicine, The University of Hong Kong, Pokfulam, Hong Kong; ^4^Graduate School, Beijing University of Chinese Medicine, Beijing 100029, China

## Abstract

Diabetic cardiomyopathy is one of the major complications among patients with diabetes mellitus. Diabetic cardiomyopathy (DCM) is featured by left ventricular hypertrophy, myocardial fibrosis, and damaged left ventricular systolic and diastolic functions. The pathophysiological mechanisms include metabolic-altered substrate metabolism, dysfunction of microvascular, renin-angiotensin-aldosterone system (RAAS) activation, oxidative stress, cardiomyocyte apoptosis, mitochondrial dysfunction, and impaired Ca^2+^ handling. An array of molecules and signaling pathways such as p38 mitogen-activated protein kinase (p38 MAPK), c-Jun N-terminal kinase (JNK), and extracellular-regulated protein kinases (ERK) take roles in the pathogenesis of DCM. Currently, there was no remarkable effect in the treatment of DCM with application of single Western medicine. The myocardial protection actions of herbs have been gearing much attention. We present a review of the progress research of herbal medicine as a potential therapy for diabetic cardiomyopathy and the underlying mechanisms.

## 1. Introduction

Diabetic cardiomyopathy (DCM) resulted from diabetes mellitus ultimately leads to heart failure, increasing the mortality in diabetic patients. There was a 2.4-fold increase in the risk of heart failure in the male diabetic subjects and fivefold in the female diabetic subjects [1]. DCM is characterized by left ventricular hypertrophy, myocardial fibrosis, and compromised left ventricular systolic/diastolic function [2]. Currently, there is still lack of feasible therapeutic approach for DCM. The Chinese traditional medicine has a long history in the treatment of glucose metabolism disorder and cardiovascular disease. Most recently, Li et al. [3] uncovered the antidiabetes effect of artemisinins, and this finding has been published in the Journal of cell. Using herbs to treat many chronic diseases such as diabetes and its complication has been recognized by an increasing number of scientists and clinic physicians for their multitarget effect and comprehensive source. In this review, we discuss the molecular mechanisms for the pathogenesis of DCM and then the study progress of herbal medicines as potential therapeutic agents for DCM.

## 2. Pathophysiological Mechanisms of DCM

**Figure 1:**
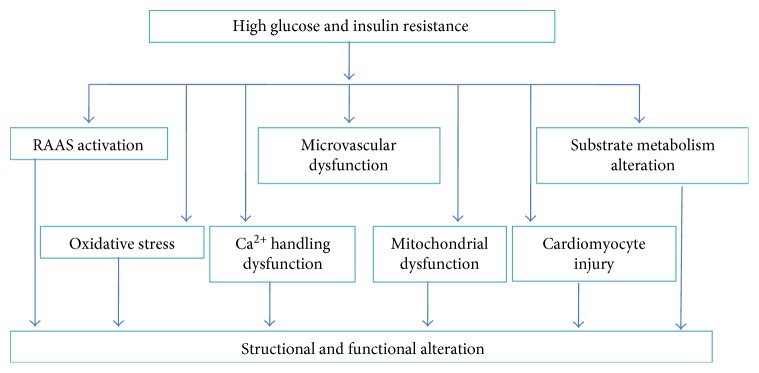
Pathophysiological mechanisms for DCM. RASS: renin-angiotensin-aldosterone system; DCM: diabetic cardiomyopathy.

The function and structure of the heart are altered with the development of DCM. The left ventricular diastolic dysfunction is one of the pathological features of DCM, which occurs in isolation and precedes the development of systolic dysfunction [4]. The ventricular hypertrophy and myocardial fibrosis are the structural alteration of DCM. Hyperglycemia, insulin resistance, microcirculation dysfunction, and neurohormone activation are pathological triggers for DCM. The hyperglycemia and insulin resistance are responsible for altered substrate metabolisms including increased free fatty acid (FFA) oxidation, intramyocardial triglyceride accumulation, and reduced glucose utilization. The altered substrate metabolisms contribute to morphological changes of cardiomyocytes. Moreover, oxidative stress, the dysfunction of mitochondrion, abnormalities in Ca^2+^ homeostasis, and cardiomyocyte apoptosis all promote DCM development (Figure 1).

### 2.1. Neurohormone Activation and Microcirculation Dysfunction

Neurohormone activation is characterized by upregulation of sympathetic nervous system and renin-angiotensin-aldosterone system (RAAS). Circulating Ang II, the atrial natriuretic peptide (ANP), B-type natriuretic peptides (BNP), and catecholamines, as well as endothelin (ET-1) both in intramyocardium and circulating, are significantly increased in the context of hyperglycemia. Activation of RAAS and elevated ET-1 contribute to the myocardial fibrosis. ET-1 is responsible for vasoconstriction and myocardial ischemia. Endothelia-derived NO, an endogenous vasodilator, is remarkably decreased in the diabetic status. Impaired microvascular blood flow, sustained hyperglycemia, and excess generation of reactive oxygen species (ROS) are contributors for endothelial dysfunction.

### 2.2. Altered Substrate Metabolism

Substrate metabolism changes in diabetes are triggered by hyperglycemia and insulin resistance. In diabetes, the myocardial glucose utilization is significantly reduced for the depletion of glucose transporter proteins 1 (GLUT-1) and GLUT-4, resulting in the diabetic rely almost exclusively on FA as an energy production source [5]. Elevated circulating and cellular FFAs are attributable for increased adipose tissue lipolysis and hydrolysis of accumulated myocardial triglyceride. FFAs can inhibit glucose oxidation by activating peroxisome proliferator-activator receptor-*α* (PPAR-*α*), which increases the expression of pyruvate dehydrogenase kinase 4 (PDK4) involved in regulating enhanced mitochondrial FA uptake and reducing glucose oxidation. Moreover, elevated circulating and cellular FFAs can enhance peripheral insulin resistance. In the long term, increased myocardial FA utilization leads to lipotoxicity to the cardiomyocytes, characterized by myocyte lipid accumulation, mitochondrial dysfunction, increased oxygen demand, and excessive generation of ROS [6, 7]. Collectively, the enhanced peripheral insulin resistance, reduced cellular glucose utilization, and lipotoxicity are responsible for the cardiomyocyte injury and myocardial remodeling.

### 2.3. Oxidative Stress

In the physiological state, the ROS is eliminated by antioxidant system. However, in the diabetic settings, excessive production of ROS is responsible for oxidative stress and correlates with the development of DCM for its ability to damage proteins and DNA and lipid membranes. In the diabetic heart, ROS are derived from mitochondrial source, nicotinamide adenine dinucleotide phosphate (NADPH) oxidase, and uncoupled NO synthases (NOS). The role of NADPH oxidase is the most important of the three sources in the development of DCM. Tumor necrosis factor-*α* (TNF-*α*) could induce cardiomyocyte hypertrophy by triggering the activity of NADPH oxidase [8]. Moreover, ROS derived from NADPH oxidase promotes the myocardial interstitial fibrosis and the mechanism involving increased activation of matrix metalloproteinases (MMP), expression of profibrotic genes, and activation of NF-*κ*B [9]. As previously illustrated, mitochondrion is another major source for ROS production. The mitochondria themselves are susceptible to the ROS they produce, leading to local damage to mitochondrial DNA and membranes, generating more ROS as a result of a positive feedback mechanism [10].

### 2.4. Impaired Ca^2+^ Metabolism

Under the physiological state, Ca^2+^ influx induced by the activation of voltage-dependent L-type Ca^2+^ channels and then triggers the release of Ca^2+^ stored in the sarcoplasmic reticulum via ryanodine receptors (RyR) through a Ca^2+^-induced Ca^2+^-release mechanism. Free Ca^2+^ binds to troponin C and results in cardiomyocyte contraction. [Ca^2+^] is pumped out of cytosol and returns to a diastolic level mainly by the activation of the sarcolemmal Na^+^/Ca^2+^ exchanger, the sarcoplasmic reticulum Ca^2+^-ATPase2a (SERCA2a), and the sarcolemmal Ca^2+^-ATPase [11]. In the diabetic state, the decreased activity of SERCA results from the interaction of advanced glycation end products (AGE) with SERCA and the overexpression of SERCA2a inhibitor phospholamban (PLB) and could be reversed by insulin treatment [11]. Suppression of SERCA2a ultimately leads to Ca^2+^ overload in the cytosol and diastolic dysfunction [12]. The activity and expression of Na^+^/Ca^2+^ exchanger which contribute to the removal of [Ca^2+^] is also decreased in the diabetic state. The abnormal function of RyR induced by AGE/RAGE and oxidative stress contributes to sarcoplasmic reticulum Ca^2+^ leak, decreased sarcoplasmic reticulum-stored Ca^2+^, and systolic Ca^2+^ transient [13, 14]. Moreover, the decreased ATP synthesis rates which result from the reduced uptake of Ca^2+^ by mitochondria cause impaired contractility ability. Consequently, the disorder of Ca^2+^ handling contributes to diastolic/systolic dysfunction and left ventricular hypertrophy.

### 2.5. Cardiomyocyte Injury

Cardiomyocyte injury manners include apoptosis, necrosis, and autophagy. Apoptosis results from oxidative stress, mitochondrial dysfunction, and abnormalities in Ca^2+^ handling. Cardiomyocyte necrosis results in interstitial collagen deposition and ultimately leads to myocardial fibrosis [15].

Autophagy, a “housekeeping” subcellular process that maintains the cell nutrition homeostasis and self-renewal by degrading damaged proteins and organelles, is an alternate form of programmed cell death. Autophagy is also considered as a process that maintains cell survival in the condition of starvation and other cell stressors, for its regulation in the turnover of long-lived proteins, and protects cells. However, the dysregulated autophagy may result in excessive cell death. The role of autophagy in pathogenesis of DCM remains controversial. Xie et al. [16, 17] reported that a low constitutive autophagy is essential for protecting cardiomyocytes from hyperglycemic damage, whereas, the defect autophagy in diabetes contributes to the development of DCM. Coincidence with this finding, Zhao et al. showed that enhanced autophagy prevents DCM induced by STZ administration [18]. However, Hou et al. showed that AGE impairs the cell viability of rat neonate cardiomyocytes in a dose-dependent manner by inducing autophagy [19]. Collectively, well-regulated autophagy is essential for DCM attenuation.

## 3. Molecules and Signaling Pathways

**Figure 2:**
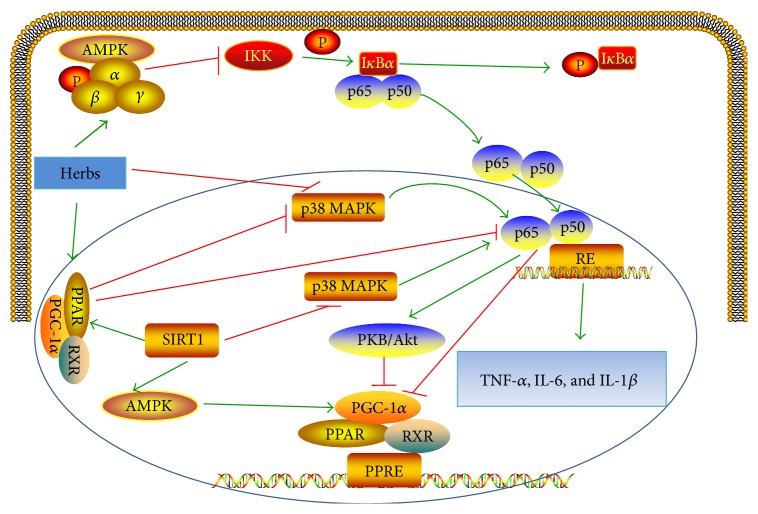
Inflammation cascade in pathogenesis of DCM. AMPK: AMP activated-protein kinase; MAPK: mitogen-activated protein kinase; SIRT1: sirtuin 1; IKK: I*κ*B kinase; RE: response element; PPAR: peroxisome proliferator-activated receptor; PGC1*α*: PPAR-*γ* co-activator-1; RXR: retinoid X receptor; PPRE: PPAR response elements; TNF-*α*: tumor necrosis factor-*α*; IL: interleukin.

**Figure 3:**
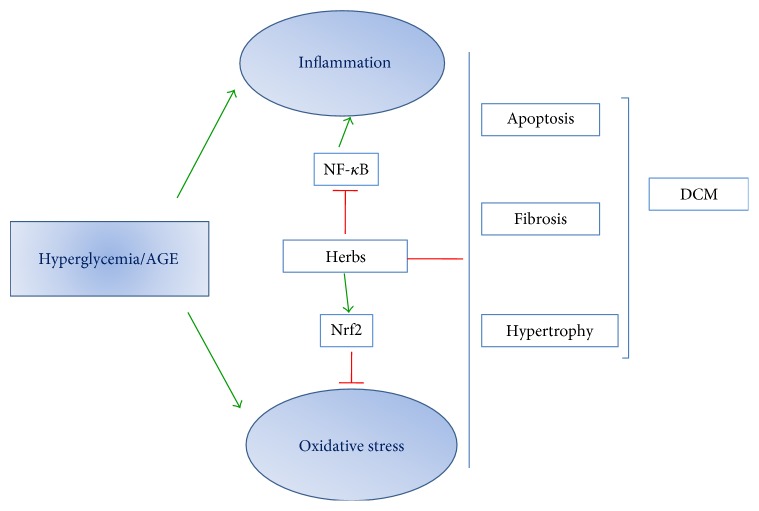
Potential Mechanisms of herbal medicine protects against diabetic cardiomyopathy. Nrf2: transcription factor NFE2-related factor 2.

NF-*κ*B is a key transcription factor that regulates inflammatory and cardiomyocyte injury processes. NF-*κ*B consists of five members including p65 (RelA), RelB, c-Rel, NF-*κ*B1 (p50 and its precursor p105), and NF-*κ*B2 (p52 and its precursor p100). The most abundant form of the NF-*κ*B family is the p65/p50 heterodimer. In resting cells, NF-*κ*B is inactive by binding to I*κ*B*α* in the cytoplasm. After high-glucose stimulation, the I*κ*B*α* is phosphorylated by I*κ*B kinase (IKK) complex, leading to the translocation of NF-*κ*B to the nucleus and binding to NF-*κ*B response element (RE) [20]. AMP-activated protein kinase (AMPK) suppresses NF-*κ*B cascade through inhibition of IKK and decreased I*κ*B*α* degradation. NF-*κ*B cascade may be induced by phosphorylation of mitogen-activated protein kinase (MAPK). It has been also showed that signal transducer and activator of transcription (STAT) may contribute to activation of NF-*κ*B and maintenance of NF-*κ*B activity. PPAR has the ability to downregulate NF-*κ*B activity by the interaction with the p65 subunit or inhibition of MAPK phosphorylation. Sirtuin 1 (SIRT1) inhibits NF-*κ*B by inhibiting the MAPK or by increasing the interaction between PPAR and p65 subunit of NF-*κ*B. SIRT1 activation leads to AMPK activation and deacetylation of PPAR-*γ* co-activator-1 (PGC1*α*). NF-*κ*B decreases the activity of PGC-1*α* directly or indirectly by activating PKB/Akt. The activation of NF-*κ*B leads to the increased proinflammatory cytokines, such as TNF-*α*, interleukin (IL)-6, and IL-1*β*, which contribute to the activation of profibrotic transforming growth factor beta (TGF-*β*) pathway. The NF-*κ*B cascade is detailed in Figure 2. Herbs inhibit the activation of NF-*κ*B by regulating AMPK, SIRT1, Akt, PPAR-*γ*, and MAPK cascades (Figure 2). Furthermore, herbs act on NFE2-related factor 2 (Nrf2), a master regulator of inflammation and oxidative status (Figure 3).

### 3.1. Mitogen-Activated Protein Kinase (MAPK) Cascade

Accumulated evidences have demonstrated that MAPK cascade including extracellular signal-regulated kinase 1/2 (ERK1/2), c-Jun N-terminal protein kinase (JNK), and p38 MAPK are involved in the diabetic complications [21]. p38 MAPK consists of four isoforms including p38*α*, p38*β*, p38*γ*, and p38*δ*. P38*α* is the major form expressed in a healthy heart, and p38*β* displays lower expression. p38 MAPK especially p38*α* MAPK contributes to the development of DCM owing to inflammation, oxidative stress, apoptosis, hypertrophy, metabolic abnormalities, and disordered Ca^2+^ handling. High glucose promotes the expression of protein kinase C (PKC) in the neonatal rat cardiomyocytes, leading to the upregulation of ROS, MAPK, and NF-*κ*B [22]. ROS activates p38 MAPK, which in turn, promotes the production of ROS; alternatively, downregulation of p38 MAPK can inhibit ROS generation and oxidative stress [23]. p38*α* MAPK has been shown to promote the cardiomyocyte apoptosis by activating STAT1 and NF-*κ*B and contribute to cardiomyocyte hypertrophy through activating the GATA4 transcription factor. MAPKPK-2 (MK2), a p38 MAPK downstream target, is responsible for downregulation of SERCA2a, FFA accumulation, and NF-*κ*B activation in the development of DCM. On the contrary, the antiapoptotic function of p38*β* MAPK in DCM has been reported [21].

ERK1/2 signaling pathway is also known as the Ras-Raf-MEK-ERK cascade. ERK1/2 pathway is triggered by the activation of Ras at the myocyte membrane. The detrimental effects of ERK1/2 in a diabetic heart are manifested as oxidative stress, apoptosis, hypertrophy, and myocardial fibrosis. ERK1/2 activated by high glucose is associated with cardiac hypertrophy in DCM [24]. ROS and ET-1 stimulate the ERK1/2 cascade; hence, antioxidant agents block the ROS generation and ERK1/2 activation as well as cardiac hypertrophy [25]. The hyperglycemia-induced increased TGF-*β* is suppressed by ERK1/2 inhibitor U0126, suggesting that ERK1/2 mediates upregulation of TGF-*β*, closely related to cardiomyocyte fibrosis [26]. Interestingly, Zhang et al. showed that fibroblast growth factor 21 (FGF21) protects the diabetic heart from cardiac apoptosis, remodeling, and dysfunction by activating ERK1/2, and this protection effect could be abolished by ERK1/2 inhibitor PD98059 [27]. The antiapoptotic or proapoptotic effect of ERK1/2 is mainly dependent on the downstream effector activities. Taken together, ERK1/2 cascade is a two-edged sword in the development of DCM [28].

JNKs, members of the family of MAPKs, mediate inflammation and cell apoptosis. Tsai et al. [29] showed that hyperglycemia enhanced NADPH oxidase-derived superoxide generation, promoting activation of JNK and NF-*κ*B activation as well as subsequent apoptosis of cardiomyocytes. JNK inhibitor and NF-ΚB siRNA abolished NF-ΚB-mediated inflammation and high-glucose-induced cardiomyocyte apoptosis.

### 3.2. AMPK Cascade

**Figure 4:**
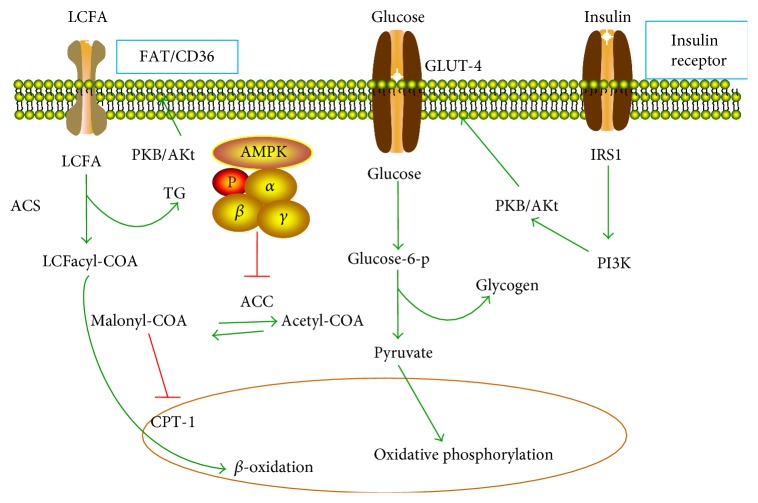
Metabolic regulation for normal heart. LCFA: long-chain fatty acids; FAT/CD36: translocation of FA transporter; GLUT: glucose transporter proteins; ACC: acetyl-COA carboxylase; ACS: acyl-CoA synthetase; CPT1: carnitine palmitoyltransferase-1; PI3K: phosphatidylinositol 3-kinase; IRS-1: insulin receptor substrate 1.

AMPK, consists of *α*, *β*, and *γ* subunits, is an important regulator of insulin signaling, cardiac energy homeostasis, and oxidative stress. The phosphorylation at T172 of the *α* subunit is well-known mechanisms for AMPK activation [30]. AMPK is upregulated in response to an enhanced AMP/ATP ratio in a stressed cellular state [31]. Both liver kinase B1 (LKB1) and Ca^2+^/calmodulin-dependent protein kinase kinase (CAMMKK) are responsible for phosphorylation at T172 of AMPK *α* subunit in the cardiomyocytes under the condition of energy depletion [32, 33]. The activated AMPK facilitates the uptake of glucose by promoting myocardial GLUT-4 expression and translocation to the plasma membrane in a similar way to insulin [34]. Furthermore, AMPK increases FA uptake by cardiomyocytes via regulating the translocation of FA transporter (FAD/CD36) to the plasma membrane [35]. Carnitine palmitoyltransferase-1 (CPT-1) is responsible for the transportation of FAs into the mitochondria for *β*-oxidation. AMPK increases FA oxidation by inducing the phosphorylation-mediated inhibition of acetyl-COA carboxylase (ACC), leading to subsequent downregulation of malonyl-COA level, an inhibitor of CPT-1. Therefore, AMPK increases the energy production by increasing glucose uptake, FA oxidation, and glycolysis. However, AMPK inhibits lipolysis by inducing the phosphorylation of hormone-sensitive lipase [36]. The metabolic regulation by AMPK is illustrated in Figure 4.

AMPK exerts an antioxidant effect by regulating the activation of Nrf2, another transcription factor that protects cardiomyocyte against oxidative stress. AMPK inhibits the NF-*κ*B cascade by inhibiting IKK activity and I*κ*B*α* degradation, or by activating downstream targets such as SIRT1, Forkhead box O (FOXO), and cardiac-enriched PGC1*α* [37, 38].

As previously illustrated, basic level of autophagy prevents against the apoptosis of cardiomyocytes. It has been demonstrated that AMPK directly activates ULK1, a homologue of yeast ATG1, through phosphorylation of Ser 317 and Ser 777 [39], or indirectly activates ULK1 by suppression of mammalian target of rapamycin complex 1 (mTORC1), resulting in enhanced autophagy [40]. Furthermore, AMPK regulates autophagy by activating FOXO, which upregulates expression of autophagy markers Bnip3, LC3, and ATG12 [30]. Recently, He et al. [41] reported that under starvation conditions, JNK1 activation leads to phosphorylation of BCL2 and dissociation of the Beclin1-BCL2 complex. However, diabetes inhibits the AMPK activity, suppresses its JNK1-BCL2 cascade, and promotes the interaction between Beclin1 and BCL2. Transfection of H9c2 cells with active JNK1 plasmid promotes BCL2 phosphorylation and disrupts the interaction between Beclin1 and BCL2, resulting in restoration of autophagy and reducing H9c2 cell apoptosis exposure to high glucose. Metformin, a well-known AMPK agonist, reduces apoptotic cell death and preserves the cardiac function by enhancing autophagy, and these effects were abolished by JNK1 inhibitor SP600125. These findings suggest that MAPK8/JNK1-BCL2 signaling is a new mechanism by which AMPK regulates autophagy [42].

### 3.3. PPAR Cascade

PPARs are responsible for the regulation of metabolism and inflammation. The subtypes of PPARs include PPAR *α*, PPAR *β*/*δ*, and PPAR-*γ*. Activation of PPARs is followed by the formation of heterodimers with the retinoid X receptor (RXR). Heterodimerization recruits PGC-1*α* and then binds to DNA-specific sequences called PPAR response elements (PPRE) and, consequently, allows the target gene transcription (CPT-1, FAT/CD36, PDK4, and GLUT-4 et al.). Of the three isoforms, PPAR *α* and PPAR *β*/*δ* express at high levels in the heart, while PPAR-*γ* enriched in adipose tissue shows a lower expression. According to Finck et al. [43] myocardial FA oxidation rates increased while glucose uptake and oxidation decreased in MHC-PPAR *α* mice, accompanied by the ventricular hypertrophy and systolic ventricular dysfunction. The MHC-PPAR *α* mice displayed metabolic phenotype and structure alteration similar to that of the diabetic heart. Burkart et al. [44] further showed that in contrast to MHC-PPAR *α* mice, MHC-PPAR *β*/*δ* mice did not develop cardiomyopathy, even in the context of a high-fat diet, owing to upregulation of GLUT-4 and enhanced rate of myocardial glucose uptake and utilization but without increased FA oxidation. Interestingly, in their study, both FAT/CD36 expression and GLUT-4 mRNA levels increased in MHC-PPAR-*γ* mice, suggesting that PPAR-*γ* shares the characteristic with both PPAR *α* and PPAR *β*/*δ* in regulation metabolism in diabetic hearts. Therefore, their findings provide the evidence that selective activation of PPAR *β*/*δ* is a promising therapeutic strategy for DCM. PGC-1*α* is the coactivator of PPARs enriched in the myocardium. PGC-1*α* regulates the mitochondrial biogenesis, FA oxidation, and glucose oxidative metabolism. PDK4 is a downstream molecule of PGC-1*α*. Selective overexpression of PDK4 in the heart of mice results in a remarkable decrease in glucose oxidation and an increase in FA oxidation. Overexpression of PGC-1*α* is associated with heart failure linked to increased FA oxidation. However, knockdown PGC-1*α* also leads to heart failure due to depletion of energy production in mitochondrion. According to Botta et al. [45] short-time exercise attenuated DCM in aged diabetic heart in db/db mice by activating PGC-1*α*. Wang et al. [46] showed that excise ameliorated DCM through activation of PGC-1*α* and Akt signaling.

All the three isoforms of PPARs exert anti-inflammation in the development of DCM for physical interaction with the p65 subunit of NF-*κ*B and inhibit the activation of certain members of the MAPK signaling pathway [47–49]. Enhanced physical interaction between p65 and PGC-1*α* contributes to the decreased activation of PGC-1*α*.

### 3.4. Phosphatidylinositol 3-Kinase (PI3K)/PKB/Akt Cascade

PI3K takes a crucial role in insulin pathway and cardiac adaptation including protein synthesis, FA and glucose metabolisms, and cell survival regulation. Activation of PI3K subsequently targets to the upregulation of downstream effectors including PKB/Akt, glycogen synthase kinase (GSK)-3Β, and mTOR. The activation of PKB/Akt increases the uptake of glucose by inducing the translocation of the GLUT-4 protein to the cell membrane (Figure 4). However, PI3K can induce the cardiac glycogen synthesis by inhibition of PKB/Akt downstream effector, GSK-3Β. PI3K also has the ability to increase myocardial FA oxidation by promoting FAT/CD36 translocation to the sarcolemma in adult cardiomyocytes (Figure 4). PI3K stimulates autophagy by inhibiting mTORC1. NF-*κ*B indirectly activates the PKB/Akt pathway, which phosphorylates PGC-1*α* and reduces its transcriptional activity.

### 3.5. SIRT1 Cascade

SIRT1, a class III (nicotinamide adenosine dinucleotide) NAD-dependent histone deacetylase, modulates AMPK activity by deacetylating LKB1 to induce its intracellular localization [50]. SIRT1 has the ability to promote the transcriptional activity of PGC-1*α*. SIRT1 inhibits NF-*κ*B by enhancing the physical interaction between PPAR and p65 subunit or by inhibiting the phosphorylation of p38 MAPK [51]. Furthermore, Sulaiman et al. showed that upregulation of SIRT1 restores the SERCA2*α* gene expression in the context of hyperglycemia and improves the function left ventricular [52]. Taken together, SIRT1 prevents against the heart from diabetic injury by attenuating inflammation signaling cascade and improving Ca^2+^ handling [53].

### 3.6. Nrf2 Cascade

Under physiological conditions, Nrf2 locates in the cytoplasm and binds to its inhibitor kelch-like ECH-associated protein 1 (keap1). Under the condition of oxidative stress and high glucose, Nrf2 releases from keap1 and translocates into the nucleus to bind to antioxidant-responsive elements (AREs), leading to the expression of antioxidant enzymes such as NADPH quinone oxidoreductase (NQO1), heme oxygenase-1 (HO-1), superoxide dismutase (SOD), and catalase (CAT). He et al. [54] investigated the protection role of Nrf2 in the development of DCM using Nrf2-KO mice. There was an increased level of ROS in the cardiomyocytes of Nrf2-KO mice, and high glucose further increased the ROS generation in concentration and time-dependent manners.

Zhao et al. [18] have demonstrated that HO-1 prevents cardiac dysfunction by promoting the phosphorylation of AMPK and increasing the autophagy marker LC3II and Beclin1 expression in the STZ-induced diabetic heart. Activators target to Nrf2 are capable of protecting the heart from high-glucose injury.

### 3.7. MicroRNAs

miRNA is a class of conserved 19–25 nucleotide-noncoding RNAs that regulate gene expression posttranscriptionally. Recently, researchers have demonstrated that miRNAs play important roles in diabetes and related complications (Table 1). The miR-144 mimics enhance the generation of ROS and apoptosis in cardiomyocyte exposure to high glucose, which could be attenuated by an activator of Nrf2, Dh404. Inhibition of miR-144 results in suppressed ROS generation and cardiomyocyte apoptosis induced by high glucose, accompanied by the improved cardiac function in STZ-induced diabetic mice [55]. According to Jeyabal et al. [56], miR-9 expression was significantly reduced in high-glucose cultured cardiomyocytes and human diabetic hearts. miR-9 mimics attenuated hyperglycemia-induced ELAV-like protein 1 (ELAVL1) and inhibited cardiomyocyte apoptosis. Inhibition of miR-9 increased ELAVL1 and caspase-1 expression [56]. Zheng et al. showed that miR-195 expression was increased, and its target protein SIRT1 was decreased in STZ-induced type 1 and db/db type 2 diabetic mouse hearts. Anti-miR-195 in the heart improved myocardial function in STZ-induced mice by upregulating the activity of SIRT1 [57]. According to Liu et al., miR-21 promotes high-glucose-induced cardiac fibrosis though JAK/SAPK and p38 signaling pathway by suppression of dual specific phosphatase 8 (DUSP8) expression [58]. miR-200c expression is increased while its target molecule DUSP1 is decreased in DCM model and high-glucose-treated cardiomyocytes. Inhibition of miR-200c suppresses the expression of DUSP1, leading to decreased phosphorylation of ERK, p38, and JNK, as well as attenuating cardiomyocyte hypertrophy induced by high glucose [59]. Raut et al. showed that miR-30c overexpression attenuated high-glucose-induced cardiomyocyte hypertrophy by inhibiting the expression of cell division control protein 42 homolog (Cdc42) and p21-activated kinases (PAK1) [60]. Li et al. found that miR-30d promoted cardiomyocyte pyroptosis in DCM by direct repression of Foxo3a expression [61].

## 4. Herbal Medicines: Promising Therapeutic for DCM

Currently, a growing number of preclinical studies provide the evidences that herbal medicines are promising therapy for DCM (Tables 2 and 3). These herbs ameliorate cardiac injury of DCM owing to their antioxidant and anti-inflammation properties, via regulation of NF-*κ*B and Nrf2 pathways (Figure 3).

### 4.1. Triptolide

**Figure 5:**
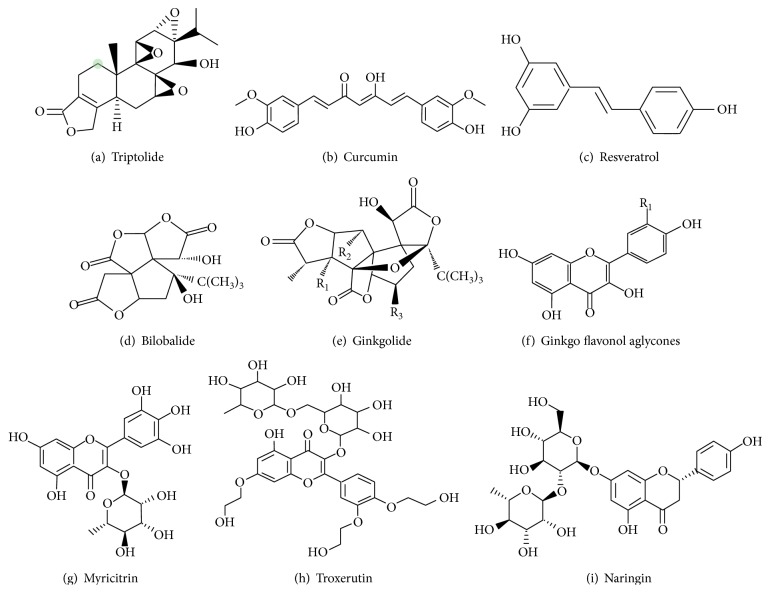
Molecular structure of the compounds described in this review.

Extracts of Tripterygium wilfordii Hook F are effective in traditional Chinese medicine for the treatment of immune inflammatory diseases including rheumatoid arthritis, systemic lupus erythematosus, and nephritis. Triptolide as the major active ingredient for Tripterygium wilfordii Hook F exerts immunosuppressive and anti-inflammatory functions [62] (Figure 5(a)). Li et al. [63] showed that triptolide at 20 *μ*g/kg/d and 100 *μ*g/kg/d attenuated the myocardial fibrosis, cardiomyocyte hypertrophy, and restored the impaired cardiac function in the rat that underwent transverse aortic constriction, associated with decreased production of profibrotic factors TNF-*α* and IL-1*β*. Wen et al. [64, 65] showed that triptolide (100, 200, or 400 *μ*g/kg/d p.o) administration for 6 weeks improved the left ventricular function of STZ-induced diabetic heart by inhibiting the expression of cardiac p38 MAPK in the upstream of NF-*κ*B activation; triptolide with dose of 200 *μ*g/kg/d displayed the best improvement. Furthermore, triptolide (20 ng/ml) attenuated inflammation of H9c2 rat cardiac cell exposure to high glucose by inhibiting NF-*κ*B activation. Guo et al. [66] showed that the left ventricle pathological structure and function of STZ-induced mice were significantly improved by triptolide (50, 100 or 200 *μ*g/kg/d p.o.) treatment for 8 weeks. The mechanism through which triptolide protects against DCM is involving inhibition of NF-*κ*B/IL-1*β* and NF-*κ*B/TNF-*α* cascades.

### 4.2. Curcumin

Curcumin, a component of turmeric found in the *Curcuma longa* plant, has been used in treating inflammatory diseases for centuries due to its antioxidant property (Figure 5(b)). Soetikno et al. [67] showed that curcumin exerts antifibrotic effect in amelioration of diabetic nephropathy owing to inhibiting PKC-*α* and PKC-*β*2, as well as the downstream cascade ERK1/2. They also demonstrated that curcumin at dose of 100 mg/kg/d for an 8-week oral administration significantly improved the left ventricular function and attenuated the progression of cardiac remodeling of STZ-induced diabetic rats by downregulating PKC-*α* and PKC-*β*2 and subsequently inactivating p38 MAPK, ERK1/2, and NF-*κ*B. Moreover, the effect of improved blood glucose of diabetic rat partly explained the decreased oxidative stress [68]. Compound (2E, 6E)-2,6-bis (2-(trifluoromethyl) benzylidene) cyclohexanone (C66) is a synthetic derivative of natural active curcumin. Pan et al. showed that pretreatment of H9c2 cells, and neonatal cardiomyocytes with C66, significantly reduced the high-glucose-induced inflammation cytokine overexpression by inhibiting NF-*κ*B. Treatment of STZ-induced diabetic mice with C66 at a dose of 5 mg/kg every other day for 12 weeks decreased the levels of plasma and cardiac TNF-*α*, endoplasmic reticulum stress, and cardiomyocyte apoptosis, as well as improved the cardiac dysfunction by inhibiting JNK phosphorylation [69, 70].

### 4.3. *Ginkgo biloba* Extract (GBE)

*Ginkgo biloba* extract (GBE) contains terpenoids, flavonoids, alkylphenols, polyprenols, and organic acids. The standardized GBE, EGb761, is pharmacologically prepared containing ginkgo flavonoids (primarily quercetin, kaempferol, and isorhamnetin) comprising 22–24% of the GBE, 6% terpenoids (3.1% ginkgolides A, B, C, and J and 2.9% bilobalide), and <5 ppm ginkgolic acid (Figures 5(d), 5(e), and 5(f)). Fitzl et al. showed that 100 mg/kg/d EGb761 orally administered for 12 weeks significantly reduced the increase of interstitial volume and collagen fibers in a diabetic rat heart [71, 72]. Furthermore, EGb761 treatment improves the hypoxia tolerance of diabetic myocardium and myocardial microvessels [73, 74]. Saini et al. [75] demonstrated that EGb761 at a dose of 50 mg/kg/d for 3 weeks significantly attenuated the index of lipid peroxidation and oxidative stress in diabetic rats and inhibited the opening of mitochondrial permeability transition pore (mPTP), ultimately leading to improvement of cardiomyopathy.

### 4.4. Resveratrol

Resveratrol (3,5,4′-trihydroxylstilbene), a natural polyphenol present in red wine and grapes, is capable to reduce blood glucose level in STZ-induced diabetic rat [76, 77] (Figure 5(c)). GLUT-4 translocation and glucose uptake are increased in STZ-induced diabetic rat myocardium by orally administrated resveratrol at a dose of 2.5 mg/kg/d for 2 weeks, the mechanism involving activation of AMPK and AKt cascades by resveratrol [78]. Resveratrol prevents high-glucose cultured neonatal rat cardiomyocyte apoptosis by inhibiting NADPH-derived ROS production and by alleviating the reduction of cardiac antioxidant enzyme activities, possibly mediated by AMPK-related signaling pathway [79]. Yar et al. showed that 10 mg/kg/d resveratrol intraperitoneal injection for 4 weeks ameliorated diabetic heart failure by increasing the expression of SIRT1 [80]. A special diet enriched with resveratrol at 0.067% (the consumption of resveratrol is estimated to be less than 100 mg/kg/d) for 12 weeks effectively restores SERCA2*α* expression and cardiac function in diabetic mice, associated with STIR1 activation [52].

### 4.5. Astragalus Polysaccharides (APS)

APS is a main active extract from the traditional Chinese medicinal herb Astragalus membranaceus. Chen et al. demonstrated that APS improved cardiac function and myocardial collagen deposition by inhibiting the local chymase-Ang II system and Ang II-activated ERK1/2 in diabetic cardiomyopathy in hamsters [81–83]. They also showed that APS can ameliorate myocardial glucose metabolism disorders in diabetic hamster by promoting expression of myocardial GLUT-4 gene and inhibiting level of PPAR *α* [84]. Pretreatment of cells with 0.8 mg/ml APS could inhibit high-glucose-induced apoptosis of H9c2 cell by decreasing the expression of caspases and release of cytochrome C from mitochondria to cytoplasm and by modulating the ratio of BCL-2 to Bax in mitochondria [85].

### 4.6. Salvia Miltiorrhiza

Salvia miltiorrhiza (Danshen), a traditional Chinese herbal medicine, is commonly used for the prevention and treatment of cardiovascular disease. According to Yu et al. [86] intraperitoneal injection Salvia miltiorrhiza 100 mg/kg/d for 4 weeks improved the heart function of diabetic rats and protected against cardiomyopathy by downregulating thrombospondin-1 (TSP-1) and TGF-*β*1 in myocardial tissue. Cryptotanshinone is an active principal ingredient isolated from Salvia miltiorrhiza (Danshen). Oral administration of 10 mg/kg/d cryptotanshinone for 28 days attenuates the cardiac fibrosis in STZ-induced diabetic rats by inhibiting STAT3 pathway and MMP-9 expression [87].

### 4.7. Flavonoids

Chrysin, a PPAR-*γ* agonist, is a natural flavonoid present in honey, propolis, and various plant extracts. According to Rani et al. [88, 89], chrysin attenuated isoproterenol-induced myocardial injury in diabetic rats by activating PPAR-*γ* and inhibiting AGE-RAGE-mediated inflammation and oxidative stress signaling pathway. Myricitrin (Figure 5(g)) is a flavone exact from the root bark of *Myrica cerifera*, Myrica esculenta, Ampelopsis grossedentata, and other plants. Zhang et al. [90] reported that pretreated AGE-cultured H9c2 cells with 25 *μ*g/ml Myricitrin for 12 h significantly decreased the AGE-induced inflammation cytokines and cell apoptosis by activating Nrf2 and inhibiting NF-*κ*B. Oral administration of Myricitrin 300 mg/kg/d for 8 weeks attenuated the cardiomyocyte apoptosis and inflammation of diabetic mice heart via regulation of AKt- and ERK-mediated Nrf2 pathways. Apigenin, a flavonoid derived in fruits and vegetables, has been shown to protect against isoproterenol-challenged diabetic myocardial injury by activation of PPAR-*γ* pathway [91]. Taxifolin is a flavonoid abound in *Pseudotsuga taxifolia*, Dahurian larch, and syn Larix dahurica Turoz. According to Sun et al. [92], Taxifolin at concentration 20 and 40 *μ*g/ml could decrease the apoptosis of high-glucose cultured H9c2 cells by inhibiting ROS generation. In vivo, Taxifolin attenuated the structure and function abnormalities by blocking NADPH oxidative activities. Troxerutin (Figure 5(h)), a bioflavonoid, protects against DCM through suppression of NF-*κ*B and JNK in a diabetic rat [93]. Hesperidin, a flavonoid isolated from citrus, has been shown to reduce oxidative stress and apoptosis and attenuate myocardial injury in isoproterenol-STZ rat via activation of PPAR-*γ* [94]. Nobiletin treatment (50 mg/kg/d p.o. 11 weeks) attenuates diabetic heart injury by suppression of oxidative stress, JNK, p38 MAPK, and NF-*κ*B pathways [95]. 80 *μ*M Naringin (4,5,7-trihydroxyflavonone-7-rhamnoglucoside, Figure 5(i)) pretreated for 2 hours protects H9c2 cells from high-glucose injury by ROS scavenging and MAPK cascade inhibiting [96]. Liquirtin, a major constituent of Glycyrrhiza radix, exerts various pharmacological activities. Liquirtin prevents myocardial injury induced by high fructose feeding by inhibiting NF-*κ*B and MAPK cascades [97, 98].

### 4.8. Ginseng

Shengmaisan, a traditional Chinese recipe, consists of Radix Ginseng, Radix Ophiopogonis, and Fructus Schisandrae. According to Zhao et al. [99], cardiac dysfunction, hypertrophy, and fibrosis in diabetic mice are improved by 4.5 g/kg daily Shengmaisan treatment for 24 weeks through suppression of TGF-*β* pathway. Ni et al. [100] showed that Shengmai powder and Danshen decoction (consists of Radix Ginseng 9 g, Radix Ophiopogonis 9 g, Fructus Schisandrae 6 g, Radix Salviae Miltiorrhizae 30 g, Lignum Santali 6 g, and Fructus Amomi 6 g) inhibited the myocardial fibrosis in the diabetic rat through inhibiting TGF-*β* and TSP-1. Sen et al. [101] showed that alcoholic ginseng root (200 mg/kg/d, daily oral gavage) for 2 or 4 months is effective in the protection of cardiomyopathy in both type 1 and type 2 diabetic mice attributed to its antioxidative and antihyperglycemia properties. According to Gu et al. [102], total saponins of *Panax ginseng* 30 mg/kg/d by gavage for 12 weeks attenuated myocardial ultrastructural injury in diabetic rat and improved the lipid profile, blood glucose, and myocardial oxidative stress level as well. The mechanism involves regulation of citric acid cycle, fatty acid metabolism, and oxidative stress. Yu et al. [103] showed that Ginsenoside Rg1 dose dependently reduced serum levels of creatinine kinase MB and cardiac troponin I and attenuated diabetic rat myocardial ultrastructural disorder. Myocardial apoptosis was reduced by Ginsenoside Rg1 associated with reduced levels of caspase-3 and increased levels of B-cell lymphoma-extra-large (Bcl-xL) in the diabetic rat myocardium.

### 4.9. Others

Broccoli sprout extract at high dose (estimate an Nrf2 activator- sulforaphane availability at 1.0 mg/kg) by gavage every other day for 3 months significantly prevents cardiac dysfunction of diabetic db/db mice by upregulating Nrf2 transcription [104]. Cardiac lipid accumulation and deposition of collagen are inhibited by 8-week oral treatment of Dendrobium officinale Kimura et Migo at dose of 75, 150, and 300 mg/kg/d [105]. Dendrobium officinale Kimura et Migo attenuates the diabetic heart injury by downregulating the NF-*κ*B-mediated inflammation cascade [105]. Flos Puerariae extract at dose of 100 and 200 mg/kg/d for 10 weeks prevents myocardial apoptosis in STZ-induced diabetic heart through inhibiting oxidative stress, associated with suppression of JNK and p38 MAPK activation [106]. Mangiferin (20 mg/kg/d p.o. 16 weeks) inhibits ROS accumulation, AGEs/RAGE production, and NF-*κ*B nuclear translocation, attenuating cardiac injury induced by STZ and high-fat diet [107]. Total aralosides of *Aralia elata* (Miq) seem (TASAES) from Chinese traditional herb Longya *Aralia chinensis* L was found to prevent diabetes-induced cardiac dysfunction and pathological damage through upregulating L-type Ca^2+^ channel current in cardiac cells and decreasing connective tissue growth factor expression at dose of 4.9, 9.8 mg/kg, and 19.6 mg/kg/d by gavage, respectively, for 8 weeks [108]. Duan et al. [109] showed that the total saponins of Aralia taibaiensis exerted cytoprotective effects against oxidative stress induced by hyperglycemia through the Nrf2/ARE pathway. Chang et al. showed that berberine, a plant alkaloid, improves insulin resistance in H9c2 cardiomyocytes partly due to stimulation of AMPK activity [110]. They further found that berberine improved cardiac function and attenuated cardiac hypertrophy and fibrosis in a high-fat diet and STZ-induced diabetic rats through activation of AMPK and Akt [111]. According to Shen et al. [112], Shensong Yangxin Capsule inhibits diabetic myocardial fibrosis via suppressing TGF-*β* pathway.

## 5. Conclusion and Future Perspectives

DCM, featured by structure and function alteration, is a multifactorial disease. Extensive preclinical studies investigated the molecular targets for pathogenesis of DCM, and identified herbs that act on these targets are potential therapeutic approaches for DCM. However, at present, most clinical studies have small sample sizes and are not performed using a randomized design, and thus hamper the application of herbal medicines in patients with DCM. The combination of the herbs with Western medicine and joint application of herbal medicine on diabetic cardiomyopathy are superior to individual applications and are still under exploring. Hence, clinical trials in high-quality are needed in the future. Furthermore, exploring potential therapeutic target will contribute to detect new herbs for the treatment of DCM. The safety and drug interaction should be paid attention to ensure the wild and effective application of herbs in the DCM treatment.

## Figures and Tables

**Table 1:** miRNA functions in DCM.

miR type	Experimental model	Mechanism	Target gene	Reference
miR-144	STZ-induced diabetic mice	Increased oxidative stress and cardiomyocyte apoptosis	Nrf2	[55]
miR-9	Human diabetic hearts, high-glucose cultured human	Prevented cardiomyocyte apoptosis	ELAVL1	[56]
miR-195	STZ-induced diabetic mice, db/db mice	Increased oxidative stress and apoptosis	SIRT1	[57]
miR-21	High-glucose cultured primary cardiac fibroblasts	Increased cardiac fibrosis	DUSP8	[58]
miR-200c	High-fat diet plus STZ-induced diabetic rat, high-glucose cultured cardiomyocytes	Decreased cardiomyocyte hypertrophy	DUSP1	[59]
miR-30c	STZ-induced diabetic rat, high-glucose cultured cardiomyocytes	Decreased cardiomyocyte hypertrophy	PAK1 and Cdc42	[60]
miR-30d	STZ-induced diabetic rat	Increased cardiomyocyte pyroptosis	Foxo3a	[61]

Nrf2: factor-erythroid 2-related factor 2; ELAVL1: ELAV-like protein 1; SIRT1: sirtuin 1; DUSP: dual specific phosphatase; PAK1: p21-activated kinases; Cdc42: cell division control protein 42 homolog; Foxo3a: Forkhead box O3.

**Table 2:** In vitro studies of herbs in the application of DCM.

Drug	Dosage	Experimental model	Reference
Triptolide	20 ng/ml	High-glucose cultured H9c2 rat cardiac cells	[64]
C66	2.5, 5, or 10 *μ*mol/L	High-glucose cultured H9c2 cells	[69]
C66	2.5, 5, or 10 *μ*mol/L	High-glucose cultured neonatal rat cardiomyocytes	[69]
Resveratrol	50 *μ*M	High-glucose cultured neonatal rat cardiomyocytes	[79]
Astragalus polysaccharides	0.8 mg/ml	High-glucose cultured H9c2 cardiomyocytes	[85]
Myricitrin	25 *μ*g/ml	AGE-induced H9c2 cells	[90]
Taxifolin	20, 40 *μ*g/ml	High-glucose cultured H9c2 cells	[92]
Naringin	80 *μ*M	High-glucose cultured H9c2 cells	[96]
Total saponins of Aralia taibaiensis	25, 50, and 75 *μ*g/ml	G/GO cultured H9c2 cardiomyocytes	[109]

C66; Compound (2E, 6E)-2,6-bis (2-(trifluoromethyl)benzylidene) cyclohexanone; AGE: advanced glycation end products; G/GO: 33 mM glucose + 15 mU glucose oxidase.

**Table 3:** In vivo studies of herbs in the application of DCM.

Drug	Dosage	Administration	Experimental model	Reference
Triptolide	100, 200, or 400 *μ*g/kg/d	p.o. 6 weeks	STZ-induced diabetic rat	[64, 65]
Triptolide	50, 100, or 200 *μ*g/kg/d	p.o. 8 weeks	STZ-induced diabetic rat	[66]
Curcumin	100 mg/kg/d	p.o. 8 weeks	STZ-induced diabetic rat	[68]
C66	5 mg/kg/d	p.o. every other day for 12 weeks	STZ-induced diabetic mice	[69, 70]
EGb761	100 mg/kg/d	p.o. 12 weeks	STZ-induced diabetic rat	[71, 72]
EGb761	50 mg/kg/d	p.o. 3 weeks	STZ-induced diabetic rat	[75]
Resveratrol	2.5 mg/kg/d	p.o. 2 weeks	STZ-induced diabetic rat	[78]
Resveratrol	10 mg/kg/d	i.p. 4 weeks	STZ-induced diabetic rat	[80]
Resveratrol	Diet enriched with resveratrol at 0.067%	p.o. 12 weeks	STZ-induced diabetic mice	[52]
Astragalus polysaccharides	1-2 g/kg/d	p.o. 10 weeks	STZ-induced diabetic hamsters	[81–84]
Salvia miltiorrhiza	100 mg/kg/d	i.p. 4 weeks	STZ-induced diabetic rat	[86]
Cryptotanshinone	10 mg/kg/d	p.o. 28 days	STZ-induced diabetic rat	[87]
Myricitrin	300 mg/kg/d	p.o. 8 weeks	STZ-induced diabetic mice	[90]
Taxifolin	25, 50, 100 mg/kg/d	p.o. 4 weeks	STZ-induced diabetic mice	[92]
Troxerutin	150 mg/kg/d	p.o. 4 weeks	STZ-induced diabetic rat	[93]
Nobiletin	50 mg/kg/d	p.o. 11 weeks	STZ-induced diabetic mice	[95]
Liquirtin	8, 16 mg/kg	p.o. 10 weeks	High fructose-induced diabetic mice	[98]
Shengmaisan	4.5 g/kg/d	p.o. 24 weeks	db/db mice	[99]
Alcoholic ginseng root	200 mg/kg/d	p.o. 2 or 4 months	STZ-induced diabetic mice and db/db mice	[101]
Total saponins of *Panax ginseng*	30 mg/kg/d	p.o. 12 weeks	STZ-induced diabetic rat	[102]
Ginsenoside Rg1	10, 15, 20 mg/kg/d	i.p. 12 weeks	STZ-induced diabetic rat	[103]
Dendrobium officinale Kimura et Migo	75, 150, 300 mg/kg/d	p.o. 8 weeks	STZ-induced diabetic mice	[105]
Flos Puerariae extract	100, 200 mg/kg/d	p.o. 10 weeks	STZ-induced diabetic mice	[106]
Mangiferin	20 mg/kg/d	p.o. 16 weeks	STZ and high-fat diet induced diabetic rat	[107]
TASAES	4.9, 9.8, and 19.6 mg/kg/d	p.o. 8 weeks	STZ-induced diabetic rat	[108]
Berberine	100 mg/kg/d	p.o. 16 weeks	High-fat diet and STZ-induced diabetic rat	[111]

EGb761: *Ginkgo biloba* extract 761; TASAES: total aralosides of *Aralia elata* (Miq) seem.
